# Removal of arsenic(V) using pure zeolite (PZ) and activated dithizone zeolite (ADZ) from aqueous liquids: application to green analytical chemistry

**DOI:** 10.1007/s44211-024-00509-7

**Published:** 2024-02-21

**Authors:** Hossam F. Nassar, Mahmoud A. Mohamed

**Affiliations:** 1 Environmental Science and Industrial Development Department, Faculty of Postgraduate Studies for Advanced Sciences, Beni-Suef University, Beni-Suef, 62511 Egypt; 2 Hikma Pharmaceutical Company, Beni-Suef, Egypt

**Keywords:** Adsorption, Activated zeolite dithizone, Arsenic(V), Isotherm, Reaction kinetics, Green analytical chemistry

## Abstract

The primary aim of the present investigation was to evaluate the efficiency of pure zeolite and activated dithizone zeolite for arsenic(V) removal from aqueous solutions. The analytical eco-scale and analytical greenness for sample preparation results confirm that the proposed method is environmentally friendly. Zeolite adsorbents were characterized and tested for their ability to adsorb arsenic(V) from wastewater. Our study delved into arsenic(V) sorption behavior on pristine and activated zeolites. Through steady-state experiments using pure zeolite and activated dithizone zeolite, we examined the sorption of arsenic from aqueous solutions. We optimized operational parameters, including pH, adsorbent dosage, contact time, and arsenic(V) concentration. Our findings revealed that the Langmuir and Freundlich adsorption isothermal models were highly influential in fitting the experimental data, resulting in statistically significant outcomes. This study highlights the potential of zeolites as outstanding adsorbents for removing arsenic(V) from aqueous solutions. The calculated maximum adsorption capacity (*q*_max_) of pure zeolite and activated dithizone zeolite was 18.2 and 21.1(mg/g), respectively, with *R*^2^ = 0.999. According to Freundlich’s linear model, the experimental isothermal data indicated that activated dithizone zeolite has a higher value of k_f_ constant and a lower value of the 1/n constant than that obtained for pure zeolite. These results imply favorable adsorption of arsenic(V) on activated dithizone zeolite.

**Graphical abstract:**

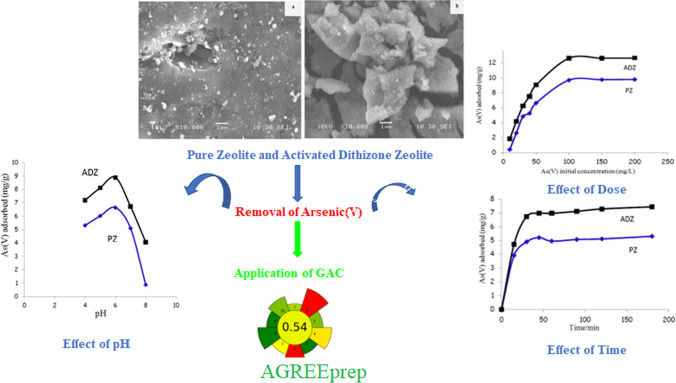

## Introduction

Heavy metal compounds have been considered one of the major water contaminants and have environmental concerns because of their high toxicity, long persistence, bioaccumulation, and negative health effects, even at very low concentration levels. Arsenic compounds are a naturally occurring element in the environment. These compounds are toxicants most often used as a pesticide. Both organic and inorganic forms of arsenic compounds can be found in organic forms of water. Nevertheless, the significance of the organic form diminishes when it undergoes a biotransformation process and detoxification through methylation. Aquatic systems contain inorganic arsenic in four distinct oxidation states. (− 3, 0, + 3, and + 5). The most used oxidation states are (+ 3 and + 5). Whereas (− 3 and 0) elemental states are extremely rare depending on the prevailing pH and redox conditions, a trivalent form of arsenic, often called arsenite, arsenic III has a charge of + 3. Considering its high solubility and ability to dissolve in groundwater, it has major implications for drinking water. Various health problems, including skin lesions, cardiovascular disease, and some types of cancer, can be linked to inorganic arsenic III compounds [1, 2]. Arsenic(V) (As(V)) compounds are ubiquitous pollutants in the environment and notoriously carcinogenic compounds, particularly in the case of humans, who primarily encounter arsenic through water and food consumption, the quantities of these chemical compounds are significant. Moreover, Arsenic compounds can be deposited from atmospheric particles onto water and soil [3, 4]. This underscores the significance of effectively and sustainably eliminating hazardous heavy metals at a reasonable cost. Hence, the utilization of various natural adsorbents, such as bentonite and activated aluminum, Zeolite, activated charcoal, clay, soya cake, wool, and functionalized activated carbon, have been employed to remove Arsenic compounds from polluted waters [5]. There is a notable inclination towards acknowledging the significance of environmentally sustainable practices within a quantitative analysis. Ensuring the safety and well-being of analysts and minimizing the ecological footprint of analytical processes are imperative factors to consider. When formulating novel methodologies, it is essential to consider the sustainability of the approach, encompassing the ramifications of solvents and waste generation. The industries have demonstrated commendable advancements in promoting sustainability and environmental responsibility, fostering a conducive environment for further endeavors to achieve substantial progress in this domain [6]. To enhance the efficacy of our analytical methodologies, it is imperative to evaluate their prospective environmental ramifications concurrently. Fortunately, several tools, such as analytical eco-scale (ESA) and analytical greenness for sample preparation (AGREEprep), can facilitate the adoption of sustainable analytical methods. By integrating these resources into our research endeavors, we can effectively advance scientific knowledge while upholding our obligation to safeguard the environment [7]. Arsenic(V) (As(V)) compounds can also be discharged into the environment through different activities and industries, such as electroplating, plastic surfaces, tanning, ceramics, and mining industries. The uptake of As(V) at low concentrations through food or water can cause various toxic symptoms like irritation, skin ulcers vomiting, bleeding, respiratory diseases, and lung cancer. Since, As(V) can easily diffuse and penetrate surface and groundwater streams leading to several hazardous effects on biota and humans [7, 8]. Hence, the Environmental Protection Agency (EPA) and the International Agency for Research on Cancer (IARC) have classified As(V) as a substance with the potential to cause cancer in humans. Different removal techniques, including electrochemical precipitation**,** chemical precipitation, filtration, evaporation, membrane, and ion exchange technologies, have been applied for treating wastewater containing As(V) compound which has the chemical formula Na_3_AsO_4_ [9]. These methods might be inefficient or cost-ineffective because of the high initial concentration of As(V) compounds. Another issue associated with conventional treatment processes is the generation of significant quantities of toxic sludge, which renders the overall disposal and treatment technologies incompatible with environmentally sustainable principles [10, 11]. Among the different removal techniques of heavy metals from water and wastewater, sorption by zeolites dithizone (ZD) has been selected in this study due to special characteristics [12], including high removal efficiency, low cost, molecular sieve, abundance, ease of application, and compatibility with the environmentally friendly processes [13, 14]. Dithizone (diphenyl thio carbazone) is used as an efficient modified material. Complex organic compounds with more than one donor atom (S and N atoms) significantly improve their sensitivity and selectivity to some metals. Moreover, the internal crystalline and complex structure of ZD indicates a high specific surface area [15, 16]. Because of the net negative charge of ZD compounds, they are considered a good cation exchanger. So, due to the characteristics mentioned earlier, the ZD structures can remove many water-soluble compounds. As(V) compounds can form oxyanions resulting in negative ions in an aqueous solution. Consequently, these ions will act on reducing the cationic exchange capacity, increasing the adsorption capacity of ZD compounds. Accordingly, the primary objective of this study was to assess the efficacy of natural ZD compounds in removing As(V) from aqueous solutions [17, 18].

The objective of our study was to assess the effectiveness of pure zeolite (PZ) and activated dithizone zeolite (ADZ) in removing As(V) from aqueous solutions. To accomplish this objective, we fine-tuned various operational parameters such as pH levels, adsorbent dosage, contact time, and As(V) concentration. The results of our study indicate that the Langmuir and Freundlich adsorption isothermal models were well-suited to the experimental data, yielding substantial outcomes. Furthermore, our methodology was based on sustainable environmental principles validated by ESA and AGREEprep evaluations. The combination of PZ and ADZ demonstrates high efficacy in removing As(V) from aqueous solutions, rendering our approach applicable to many practical methods.

## Materials and methods

### Chemicals

The process involved dissolving dehydrated sodium arsenate (NaAsO_3_) in de-ionized water to create a stock solution of As(V) with a 1000 mg/L concentration. HCl was added drop wisely for the dissolution of NaAsO_3_ and pH adjustment. Working solutions with different concentrations were freshly prepared from the stock solution for each experimental run. Using analytical grade chemicals ensured precise and reliable findings by eliminating potential variables. Hydroxylamine, hydrochloride, and de-ionized water were used to rinse and clean all used glassware.

### Preparation of activated dithizone zeolite adsorbent

In a 500 mL flask, the activated dithizone zeolite was prepared by refluxing 4 g natural zeolite with 1 g dithizone in about 100 mL toluene. The mixture underwent heating and stirring for 4 h, during which the temperature of the mixture was carefully regulated to reach the boiling point of toluene, approximately 55 °C. Subsequently, the reaction mixture underwent sequential rinsing with toluene, ethanol, and distilled water, repeating the process until the distinctive color of dithizone was no longer observable in the filtrate. The solid product that was produced underwent a drying process in an oven at a temperature of 70 °C for 6 h to remove the organic impurities through volatilization. Finally, the obtained ADZ was characterized and used for an adsorption study [13, 14].

### Characterization

#### Characterization of adsorbents (PZ and ADZ)

The X-ray diffractometer (XRD) utilized in this study was the PANalytical Empyrean model from the Netherlands. To analyze the phase composition and crystallinity of ADZ, Cu-Kα radiation (*λ* = 0.154 cm^−1^) with an accelerating voltage of 40 kV was employed. The scan angle ranged from 5 to 80° with a scan step of 0.04°, and the current was set at 35 mA. The KBr pellet method and the Bruker-Vertex 70 instrument for Fourier-transform infrared spectrometry (FTIR) were used to determine the functional groups in ADZ before and after adsorption. Spectral analysis was performed over a 400–4000 cm^−1^ wavenumber range. The pH at which the net charge of ADZ is zero (pHpzc) was also determined by interpolating the data to zero electrical potential. Examining the morphology and microstructure of the ADZ was accomplished using a scanning electron microscope (SEM, Holland Philips, JSM-5800). Lastly, the Brunauer–Emmett–Teller (BET) method was used to determine the PZ and adsorbents' surface area and pore size distribution. This method measured the cumulative nitrogen adsorption at 77 K using a Micromeritics 2000 instrument [10, 13].

#### Adsorption experiment

A batch technique was used in this study to conduct adsorption experiments in triplicate at ambient temperature. They agitated the As(V) solution at 200 rpm using an orbital shaker and created a calibration curve with a range of concentrations from 50 to 300 mg/L. They then prepared an arsenic solution with a 1000 mg/L concentration and exposed it to an adsorbent with a concentration of 5.0 g/L. The adsorption solutions containing Ar(V) were prepared by diluting the stock solution to an appropriate concentration and were stored in brown and amber glass bottles. A pH meter was used to measure the pH of the adsorption solution after it had been adjusted with either 0.1 M HNO_3_ or 0.1 M NaOH. They measured the concentration of As(V) remaining after a process using an atomic fluorescence spectrometer manufactured by PS Analytical Ltd. The investigation of adsorption kinetics was conducted to remove As(V) from a solution with a pH range of 6–7 and a temperature of approximately 20 °C. As(V) was dissolved in a 50 mL solution containing a composite adsorbent concentration of 5.0 g/L and initial as(V) concentrations of (5–200) mg/L. They subjected the vials to agitation at revolutions from 0 to 180 min. A study was conducted in this study to examine the effects of various parameters on the adsorption of As(V). To achieve this, they conducted experiments under varying initial pH conditions ranging from 4 to 8. They assessed the impacts of adsorbent dosage and contact time.

## Results and discussions

### Characterization of adsorbents

**Fig. 1:**
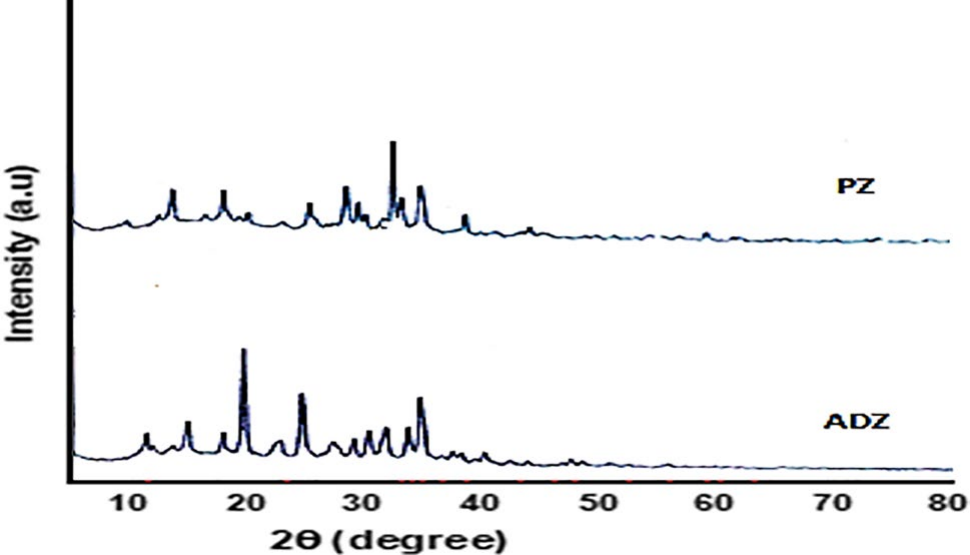
XRD patterns for PZ and ADZ

**Fig. 2:**
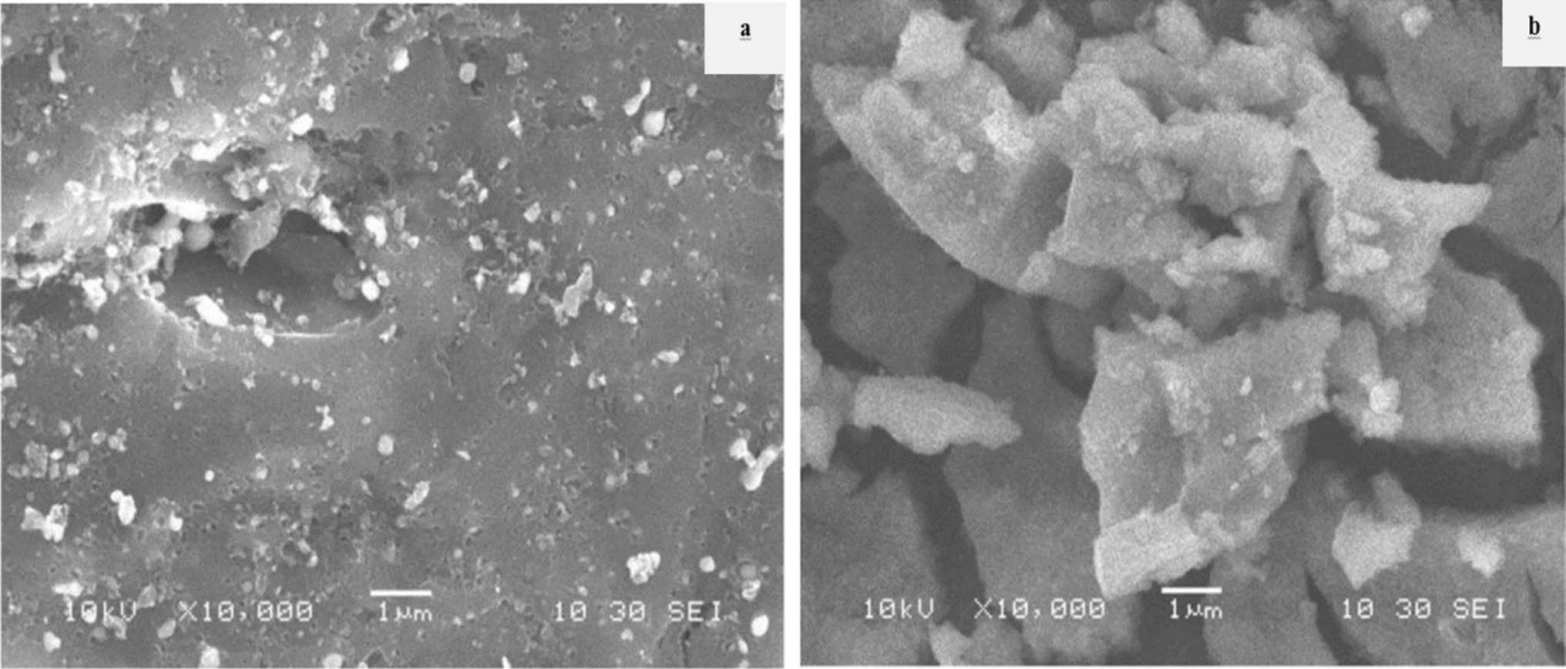
SEM micrographs of PZ (**a**) and ADZ (**b**)

**Fig.3:**
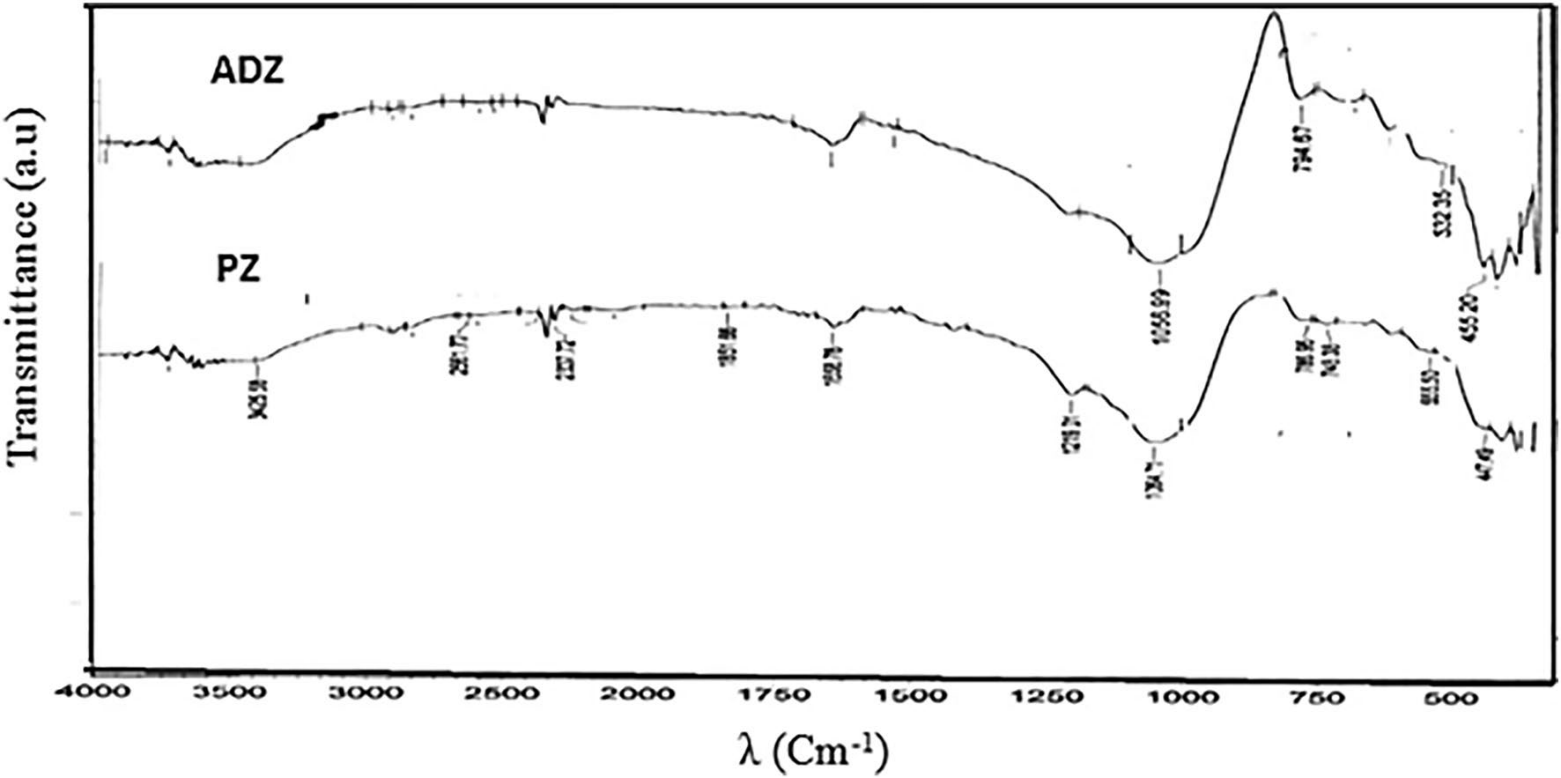
IR Spectra for PZ and ADZ

The process of X-ray diffraction (XRD) analysis is employed to ascertain the crystallinity and mineral structure of both pure zeolite (PZ) and activated dithizone zeolite (ADZ). Furthermore, the X-ray diffraction (XRD) patterns, as shown in (Fig. 1), revealed that the lattice fringe observed in the crystalline particles corresponds to the cubic face of the pure zeolite structure. Additionally, the crystallographic planes were reflected in the structure of ADZ, as reported in a previous study [13]. The diffractograms of the two purified zeolite crystal structures can be compared. There are 2 highest dithizone peaks in the ADZ diffractograms at 14.75 (*d* = 6.002 Å) and 18.93 (*d* = 4.68 Å), and several other peaks overlapping with the peak of PZ, the presence of the dithizone peak in the XRD ADZ diffractograms show that the dithizone is successfully immobilized the ADZ structure. The morphological structures of both zeolite absorbents PZ and ADZ can be performed by SEM micrographs (Fig. 2). From which, it could be observed that there are few macropores in PZ. In contrast, large aggregates were noticed in ADZ, which may indicate a higher removal rate of As(V) on ADZ than that of PZ. The characterization with FT-IR aims to determine the functional groups in (Az and ADZ) adsorbent and to see whether the As(V) has been successfully performed. FTIR spectrum of PZ and ADZ, which displays characteristics of zeolite. The zeolite framework exhibits characteristic spectral peaks within the 300–1650 cm^−1^ wave number range. Interpretation of the IR spectrum can be obtained from several absorption bands. Figure 3 shows the PZ before activation and immobilization, there is a specific wavenumber to show the functional group. The –OH group's vibration and H–O–H's bending vibration is in the range of 3425 cm^−1^ and 1627 cm^−1^ wavenumbers. Whereas the wavenumbers of 1072 cm^−1^ and 439 cm^−1^ refer to Si–O–Si and the bending vibrations of Si–O-Si. Whereas, after the PZ was successfully activated and immobilized into the ADZ, the functional group of ADZ by observing the specific wavenumber of dithizone as 1590 cm^−1^, 1450 cm^−1^, and 1312 cm^−1^ respectively for stretching vibrations CN and bending vibrations –NH, N=N bonds. Another proof that dithizone was successfully immobilized for ADZ is that there are shifts in wavenumbers of Si–O vibration from 1075 to 1090 cm^−1^ and bending vibration Si–O–Si from 480 to 495 cm^−1^ (Fig. 3). This shifting may be attributed to the interaction of Si group from PZ with the N group from dithizone. Significant microstructure changes were recorded for PZ and ADZ adsorbent, as shown in Table 1. The inclusion of dithizone in ADZ results in a reduction in the N2–BET surface area. PZ exhibits a relatively limited surface area and microporosity (1.41 nm) in comparison to ADZ (1.84 nm). The pH at which the modified ADZ indicated zero charges (pHpzc) was 7.8. The morphological structures of PZ and ADZ were analyzed using scanning electron microscopy (SEM) micrographs, as depicted in (Fig. 2a, b). These micrographs reveal a limited number of aggregates, which contribute to forming a macro pore structure in ADZ that appears brighter in comparison.

**Table 1:** Microstructure of pure zeolite (PZ) and activated dithizone zeolite (ADZ)

Sample	Surface area (m^2^/g)	Average pore diameter (nm)	Average pore volume (cm^3^/g)
PZ	341.2	1.411	0.423
ADZ	663.5	1.849	0.868

### Effect of pH

**Fig.4:**
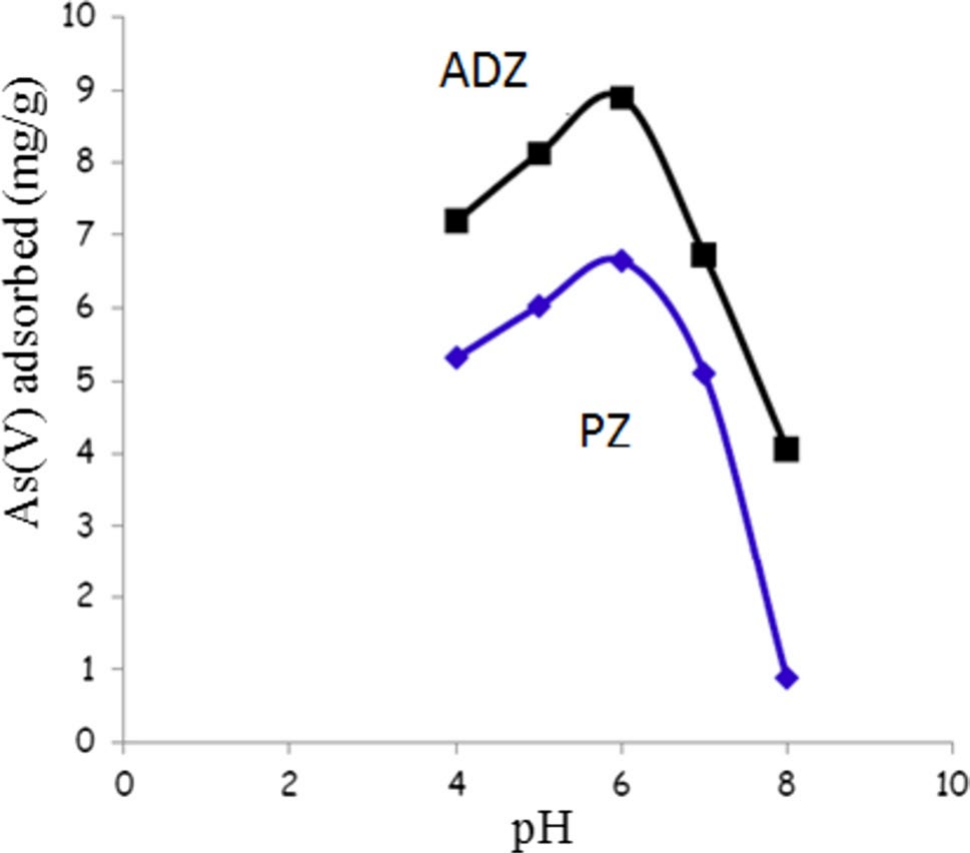
Effect of solution pH on removing As(V)

The effects of pH values ranging from 4 to 8 on the experimental outcomes of As(V) adsorption on PZ and ADZ adsorbents were studied. Figure 4 illustrates the relationship between As(V) removal percentage and the pH values ranging from 4 to 8. The data suggest that pH significantly influences As(V) removal efficiency. Additionally, it was observed that the highest adsorption levels for As(V) occurred within the pH range of 4.0–6.0. The optimal pH value for the other experiments was determined to be 6.0, as it resulted in the highest removal efficiency of As(V). The obtained data indicate that with increasing pH value from (4–6), the adsorbed amount of As(V) increased from (5–6.8) and from (7–8.9) mg/g for PZ and ADZ, respectively. Therefore, the adsorbed amount of As(V) on ADZ was much higher than that on PZ, referring to the higher removal efficiency of ADZ than PZ. Whereas, at pH > 6.0, the adsorbed amount of As(V) was sharply decreased, and consequently, the removal efficiency decreased. Based on our observations, the decrease in H^+^ ion concentration at higher pH levels may be the reason behind the phenomenon we observed. This decrease could cause competition with metal ions occupying ADZ exchange sites. This correlation is strongly linked to the point of zero charges (PZC) of ADZ, as confirmed by previous studies [19, 20]. Furthermore, we have discovered that the pH of the solution significantly impacts the performance of ADZ adsorbents. When alkaline cations like calcium, magnesium, and iron are present in the ADZ adsorbent, they can react with H^+^ ions in the medium, leading to a neutralization reaction and an increase in the pH of the solution. This increase in pH has been shown to considerably affect the removal of As(V) from the solution.

### Effect of adsorbent concentration

**Fig. 5:**
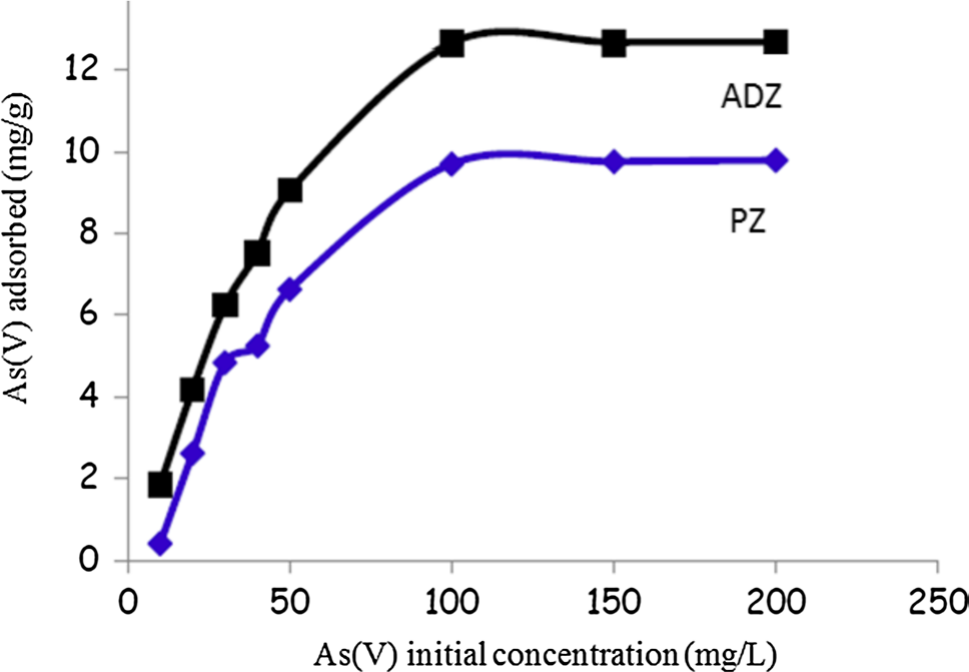
Effect of initial and adsorbed concentrations of As(V) on both (PZ & ADZ) adsorbents

The initial concentration of As(V) increased adsorbed As(V) on both adsorbents PZ and ADZ. By increasing the initial As(V) concentration from 5 to 100 (mg/g), the rate of As(V) adsorption was increased from 0.2 to 9.3 (mg/g) and from 1.9 to 12.3 (mg/g) for PZ and ADZ respectively (Fig. 5). This may attribute to at higher concentrations of the adsorbent there is a greater surface area and availability of more vacant spots and exchangeable sites that can enhance the solute to be adsorbed leading to increase in the removal efficiency [6, 17, 21, 22]. At higher adsorbent concentrations, the As(V) adsorption rate is almost constant, and the adsorption process has no significant increase. The observed effect can be attributed to the nearly complete adsorption of As(V) onto the adsorbent, as well as the attainment of equilibrium between the adsorbed As(V) and the residual unabsorbed As(V) in the solution. These findings are consistent with the results obtained by previous studies [9, 10, 23, 24].

### Effect of contact time

**Fig. 6:**
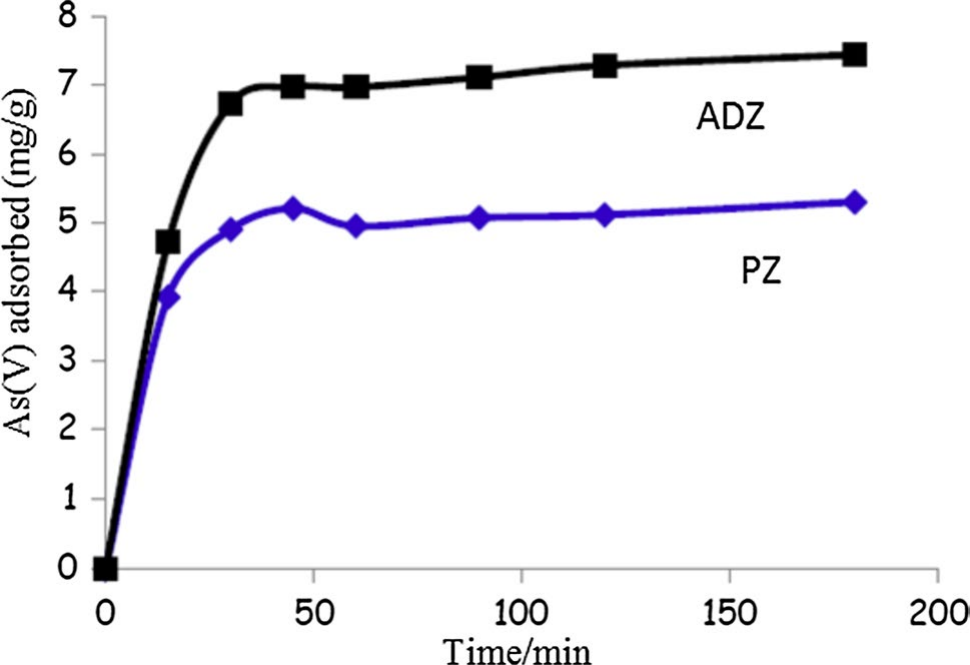
Effect of contact time on the removal of As(V)

The adsorption process is significantly influenced by contact time, which is recognized as a crucial factor. The impact of contact duration on the adsorption efficacy of As(V) onto the ADZ. The ADZ adsorbent exhibited a significant increase in the removal efficiency of As(V) from 0 to 60 min, with the highest recorded removal percentage (Fig. 6). The significant rise in numbers can be attributed to a substantial quantity of unoccupied surface sites, facilitating the adsorption process during the initial phase. Then, the removal continued to be slowly increased with increasing the contact time (60–120 min). After that, the equilibrium was achieved after 120 min. At which almost there is no significant change in the rate of As(V) removal. This can be attributed to, by the time most of the remaining vacant sites on the surface being occupied with solute. In addition, it is worth noting that the presence of repulsion forces among solute molecules located on the surface of ADZ can reduce the efficiency of the adsorption process. The results of this study were like those reported by previous researchers [17, 23, 24].

### Adsorption isotherms

**Fig. 7:**
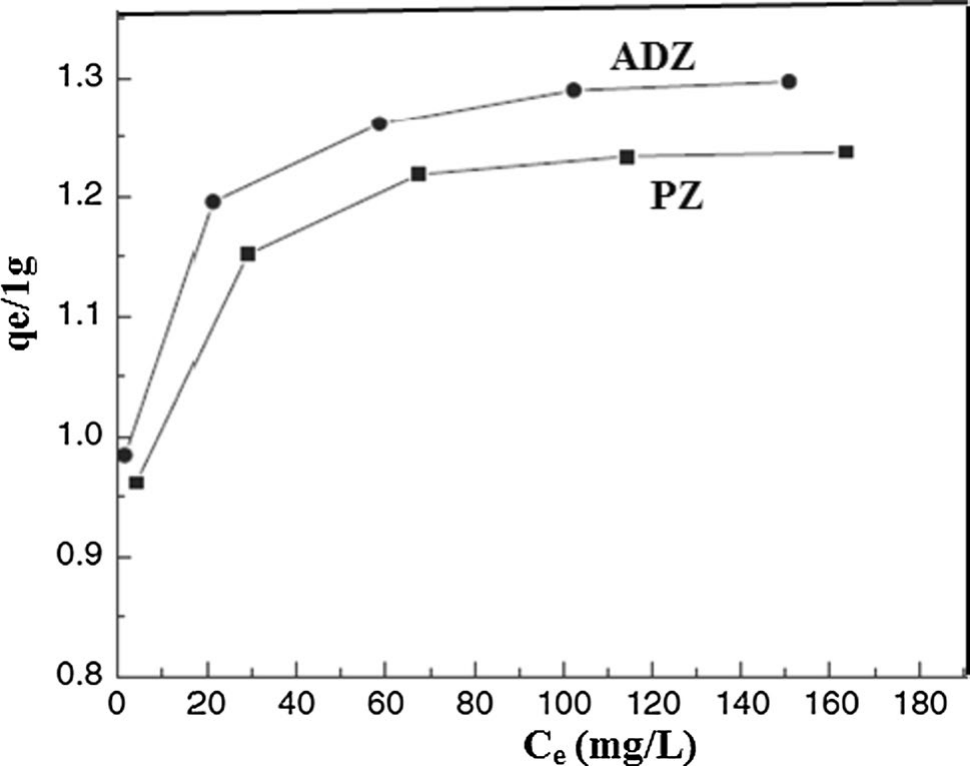
Adsorption isotherms of As(V) by PZ and ADZ

The experimental values of the As(V) adsorption isotherm were described using the Langmuir and Freundlich isotherm models. Isothermal models are commonly employed to elucidate the equilibrium distribution of adsorbed molecules between the liquid and solid phases. Figure 7 illustrates the adsorption isotherm of As(V) using both PZ and ADZ. The adsorption behavior was analyzed by examining the effects of contact time and varying concentrations of As(V). The study investigated As(V)'s adsorption characteristics on two types of adsorbents, namely PZ and ADZ. The isotherm models proposed by Langmuir and Freundlich have demonstrated a positive correlation between the equilibrium concentration of As(V) and the adsorption capacity. The Langmuir model demonstrates that As(V) adsorption on ADZ is significantly more significant than PZ, as evidenced by the data presented (Fig. 5) The Langmuir and Freundlich isotherm models can be mathematically represented by Eqs. (1 and 2), respectively, as follows:

1 $$q_{{\text{e}}} = q_{{\text{m}}} \cdot \, b \cdot c_{{\text{e}}} /\left( {1 + \, b \cdot c_{{\text{e}}} } \right),$$

2 $$q_{{\text{e}}} = \, k_{{\text{f}}} \cdot c_{{\text{e}}}^{1/n} .$$

In this context, *q*_e_ represents the sorbet amount of As(V) per gram of the adsorbent (expressed in mg/g), *q*_m_ denotes the maximum adsorption capacity (mg/g), *c*_e_ signifies the equilibrium concentration (mg/L), *b* represents the adsorption constant (expressed in L/mg), *k*_f_ denotes the adsorption capacity constants, and *n* represents the affinity constant.

The isotherm parameters in Table 2 demonstrate that the experimental data obtained were successfully fitted using the Langmuir and Freundlich linear models. The observed trend of increasing values of both *q*_m_ and *b* for As(V) adsorbed on ADZ indicates a higher adsorption efficiency of As(V) on ADZ compared to PZ. In contrast, as per the Freundlich linear model, the experimental isotherm data in Table 2 indicate that ADZ exhibits a greater *k*_f_ constant and a lesser 1/*n* constant than PZ. The findings suggest a significant affinity for As(V) adsorption on ADZ.

**Table 2:** Langmuir and Freundlich isothermal models-based parameters

Model	Adsorbents	
	ADZ	PZ
Langmuir		
*q*_m_	21.124	18.243
*b* (l/mg)	0.322	0.226
*R*^2^	0.999	0.999
Freundlich		
1/*n*	0.156	0.181
*K*_f_	10.113	8.241
*R*^2^	0.986	0.983

### Greenness assessment of methods

Several factors can affect the ecological score, such as where the sample preparation occurs (indoors or outdoors). Moreover, to achieve a high ecological score, it is important to use sustainable and renewable alternatives, minimize the use of hazardous substances, reduce waste generation to a volume of less than 10 mL, reduce energy usage, streamline processes, encourage automation, select a post-sample preparation setup that is environmentally friendly, maximize the number of samples processed per hour, and prioritize operator safety.

### ESA tool

To effectively evaluate the sustainability of a particular approach, it is imperative to employ the Eco-Scale tool, which has proven to be highly efficient. This tool considers multiple factors, including the number of reagents employed, potential risks, energy utilization, and waste production, and subsequently assigns a penalty score. The scores are subsequently combined to ascertain the comprehensive sustainability level of the approach, with a theoretical upper limit of 100. The deduction of penalty points results in a reduction of the overall score. A score on the Eco-Scale that surpasses 75 is categorized as "excellent green," whereas a score falling within the range of 50–75 is regarded as "acceptable green." A numerical value lower than 50 is considered insufficient green [25]. In the present study, the system underwent evaluation utilizing the designated tool, yielding a noteworthy eco-score of 80, thereby signifying a considerable degree of ecological sustainability. The environmental score was significantly improved by utilizing sustainable reagents such as purified water and ethanol, minimizing energy usage, and reducing waste to less than 10 mL. To obtain a thorough analysis of the penalty points, please refer to Table 3.

**Table 3:** Penalty points for the planned ESA score calculation

	Analytical eco-scale	Penalty points
Reagents	Purified water	0
	Ethanol	4
	Toluene	6
Instruments	Energy for instrument ≤ 1.5 KWh/sample	1
	Occupational hazard	0
	Oven	2
	Waste	7
	Total penalty points	20
	Eco-Scale total score	80

### AGREEprep tool

**Fig. 8:**
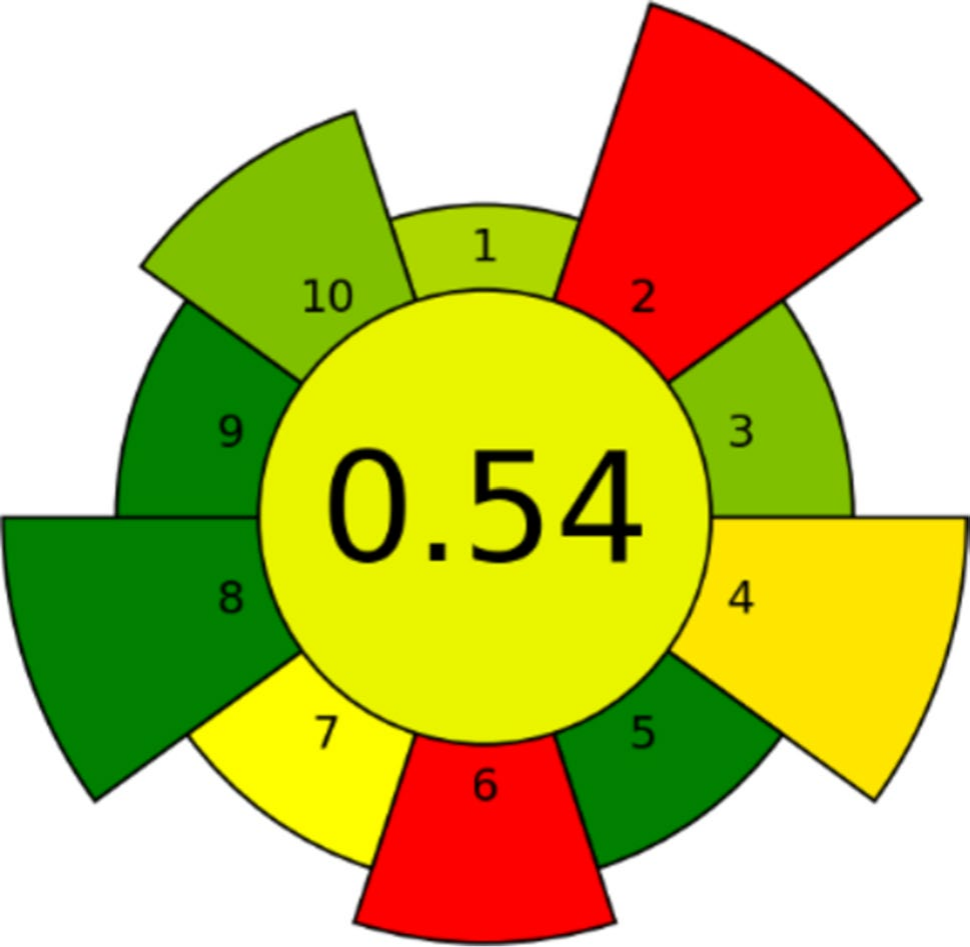
AGREEprep for the suggested approach

The attainment of robustness within analytical science is contingent upon the meticulous execution of the sample preparation procedure. The proposed methodology utilizes the AGREEprep metric, an innovative approach that assesses the environmental ramifications of various sample preparation methodologies. By incorporating this assessment procedure alongside the ten essential principles of environmentally conscious sample preparation, our system effectively showcases sustainability and a commitment to environmental preservation. The AGREEprep metric consists of ten distinct steps that evaluate an individual's proficiency. The metric utilizes a scoring system ranging from 0 to 1, where a score of 1 signifies the highest level of performance [26]. Table 4 displays graphical symbols representing the ten sectors, and we must utilize them to promote on-site sample preparation and safer solvents and reagents. We must prioritize using sustainable materials that can be used multiple times without depleting natural resources and can be replenished naturally over time. This will significantly reduce waste, help us optimize the sample processing rate, and streamline the stages involved while promoting automated processes. Additionally, we must reduce the quantities of samples, chemicals, and materials used and optimize energy efficiency. The post-sample preparation setup for analysis must be chosen carefully to ensure maximum environmental friendliness, and we must never compromise the operator's safety by following proper protocols. The findings in Fig. 8 demonstrate a numerical value of 0.54, suggesting that our methodology exhibits ecological efficiency.

**Table 4:** Criteria for score calculation of AGREEprep for the proposed method

S. no.	Criterion	Score	Weight
1	Sample preparation placement	0.66	1
	Sample preparation placement: on-line/in situ		
2	Hazardous materials	*0.0*	5
	Mass [g] or volume [mL] of problematic materials: 10		
3	Sustainability, renewability, and reusability of materials	0.75	2
	> 75% of reagents and materials are sustainable or renewable		
4	Waste	0.45	4
	Mass [g] or volume [mL] of waste: 3		
5	Size economy of the sample	***1.0***	2
	Mass [g] or volume [mL] of the sample: 0.1		
6	Sample throughput	*0.0*	3
	Hourly sample throughput: 1		
7	Integration and automation	**0.5**	2
	No. of sample prep. steps: 2 steps or fewer; degree if automation: semi-automated systems		
8	Energy consumption	***1.0***	4
	Approximate energy consumption per analysis [W]: 1		
9	Post-sample preparation configuration for analysis	***1.0***	2
	Simple, readily available detection: smartphones, desktop scanners, paper strips, etc		
10	Operator’s safety	0.75	3
	No. of distinct hazards: 1 hazard

## Conclusion

The synthesis of two specific zeolite adsorbents was conducted in this study to effectively remove As(V) from a solution. Pure zeolite (PZ) and activated dithizone zeolite (ADZ) were utilized as starting materials, and a comprehensive comparison was made between the two adsorbents. The results indicated that the ADZ adsorbent was more efficient in removing As(V) from aqueous solutions with faster adsorption kinetics. The adsorption process was explored by examining various parameters, including solution pH, contact time, and adsorbent concentration. The kinetic data and adsorption isotherm of arsenic(V) were analyzed using the Langmuir and Freundlich isothermal models, effectively demonstrating the conclusive results. It can be confidently inferred from this study that incorporating dithizone into pure zeolite has significantly improved its capacity to adsorb As(V) from contaminated water sources. ESA and AGREEprep assessments confirmed our proposed method as environmentally friendly. These results assure us that our approach is effective and sustainable. These outcomes are paramount for developing effective methods to remove As(V) from contaminated water sources.

## Data Availability

Data are available on request to the authors.
